# Mesopores induced zero thermal expansion in single-crystal ferroelectrics

**DOI:** 10.1038/s41467-018-04113-y

**Published:** 2018-04-24

**Authors:** Zhaohui Ren, Ruoyu Zhao, Xing Chen, Ming Li, Xiang Li, He Tian, Ze Zhang, Gaorong Han

**Affiliations:** 1 State Key Laboratory of Silicon Materials, School of Materials Science and Engineering, Cyrus Tang Center for Sensor Materials and Application, Zhejiang University, 310027 Hangzhou, China; 2 Center of Electron Microscope, School of Materials Science and Engineering, Zhejiang University, 310027 Hangzhou, China

## Abstract

For many decades, zero thermal expansion materials have been the focus of numerous investigations because of their intriguing physical properties and potential applications in high-precision instruments. Different strategies, such as composites, solid solution and doping, have been developed as promising approaches to obtain zero thermal expansion materials. However, microstructure controlled zero thermal expansion behavior via interface or surface has not been realized. Here we report the observation of an impressive zero thermal expansion (volumetric thermal expansion coefficient, −1.41 × 10^−6^ K^−1^, 293–623 K) in single-crystal ferroelectric PbTiO_3_ fibers with large-scale faceted and enclosed mesopores. The zero thermal expansion behavior is attributed to a synergetic effect of positive thermal expansion near the mesopores due to the oxygen-based polarization screening and negative thermal expansion from an intrinsic ferroelectricity. Our results show that a fascinating surface construction in negative thermal expansion ferroelectric materials could be a promising strategy to realize zero thermal expansion.

## Abstract (web summary)

Zero thermal expansion materials—often composites of negative and positive coefficient materials—are needed for applications that see large temperature changes. Here, the authors demonstrate that polarization around pores in single-phase mesoporous lead titanate can be used to tune thermal expansion to near zero.

## Introduction

Most of the materials expand with the increase of temperature, which is commonly referred as positive thermal expansion (PTE). Nevertheless, there exist a few zero thermal expansion (ZTE) materials exhibiting basically unchanged volume in a certain temperature range^1–4^. ZTE materials have been extensively explored because of their potential applications in high-precision instruments and devices. Not many single-phase ZTE materials are reported so far, including Invar alloys^5^, YbGaGe^6^, Mn_3_Cu_0.5_Ge_0.5_N^7^, Sc_1-x_M_x_F_3_ (M stands for Ga, Fe)^8^, and La(Fe,Si)_13_^9^. The ZTE in these materials has been mainly attributed to the specific magnetic structure^5,7,9,10^, electronic valence^6^, or local structural distortion^8^. An alternative strategy to achieve ZTE is to combine PTE and negative thermal expansion (NTE) materials^11,12^. Typical NTE materials, such as ZrW_2_O_8_^13^, ScF_3_^14^, and perovskite-type oxides (e.g., BiNiO_3_^15^ and Ca_3−x_Sr_x_Mn_2_O_7_^16^), contract with the increase of temperature, mainly due to a soft phonon mode^13,14,16^ or charge transfer^15^. Although it is widely accepted that the thermal expansion is mainly determined by the intrinsic electronic, ferroelectric, or magnetic properties that are sensitive to the microstructure of the materials^5,6,17^, the influence of surface or interface on the thermal expansion has not been fully explored. To date, a controlled ZTE in the single-phase materials has not been realized.

As a typical perovskite ferroelectric material, PbTiO_3_ (PTO) has been reported to exhibit a NTE performance with the thermal expansion coefficient (TEC) of −1.99 × 10^−5^ K^−1^ in a wide temperature range from room temperature to Curie temperature (298–763 K)^18^. Accordingly, PTO is an ideal system to explore ZTE by doping or forming solid solution states with other perovskite oxides^17–19^. An introduction of 20% La into PTO could give rise to a ZTE performance, but the temperature range was significantly suppressed due to an obvious reduction of Curie temperature^18^. More importantly, PTO-based solid solution states, such as PTO-BiFeO_3_(BFO), have been demonstrated to show a fascinating expansion behavior from NTE, PTE to ZTE by changing particle size^17^. It was revealed that the ferroelectricity is crucial for modulating thermal expansion property of PTO-based compounds^19^. Nevertheless, the ZTE and its modulation in single-phase PTO has not been reported. In this communication, we report, for the first time, a ZTE (volumetric TEC, −1.41 × 10^−6^ K^−1^, 293–623 K) of single-crystal ferroelectric PTO fibers by introducing large-scale mesopores. Microstructure characterizations show that the mesopores are faceted with two polar inner surfaces exposed. The experimental results indicate that the polarization screening within such pores is realized by the oxygen vacancies on the negative polar surface and an accumulation of oxygen occurred on the positive polar surface. The ZTE performance of PTO fibers is attributed to a synergetic effect of PTE near the mesopores due to the polarization screening and NTE from an intrinsic ferroelectricity.

## Results

### Morphology and microstructure characterization

**Fig. 1:**
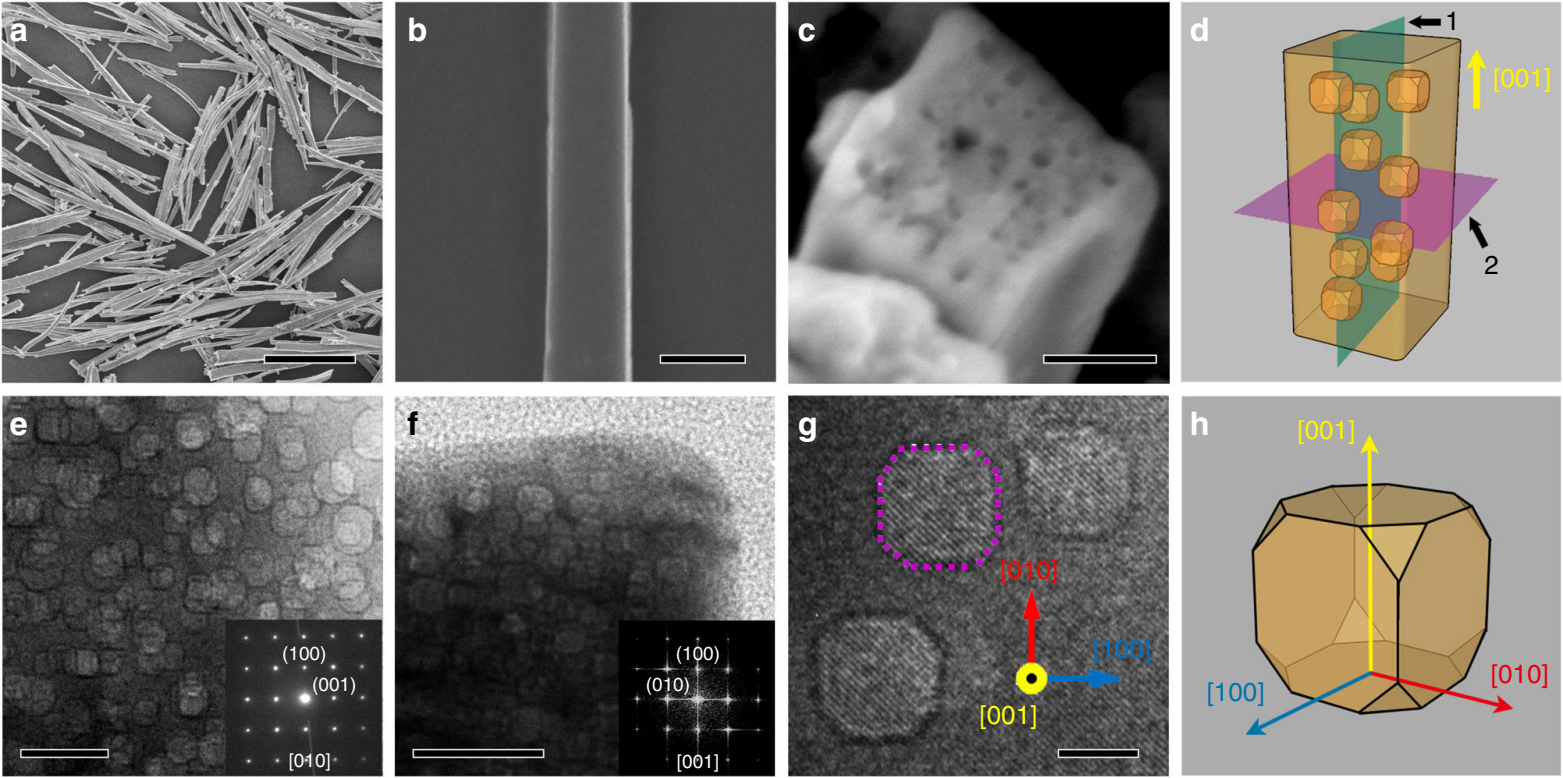
Morphology and microstructure characterization of the mesoporous PTO fibers. **a** SEM image of mesoporous PTO fibers. Scale bar, 10 μm. **b** SEM image (scale bar, 500 nm) and **c** cross-section SEM (scale bar, 100 nm) image from top view of a single fiber. **d** Schematic illustration of a mesoporous PTO fiber with many mesopores involved. **e**, **f** cross-section TEM images of mesoporous PTO fiber along radial (the blue face in **d**, marked as 1, scale bar, 20 nm) and axial (the purple face in **d**, marked as 2, scale bar, 50 nm) direction, respectively. The insets display the corresponding SAED image and FFT pattern. **g** Cross-section TEM image of several typical mesopores. Scale bar, 5 nm. **h** Truncated cube model of a single mesopore

The mesoporous perovskite PTO fibers were prepared via a solid phase transition process from pre-perovskite PTO fibers^20–22^ (Supplementary Figure 1). The ~13% density difference between pre-perovskite PTO (6.92 g cm^−3^) and perovskite PTO (7.95 g cm^−3^) has been determined via a calculation based on the unit cell parameters^20^, leading to a volume shrinking after phase transition. Moreover, highly disordered region has been discovered during the phase transition^23^, probably giving rise to a mesoporous structure in the as-prepared perovskite PTO fibers after an annealing and quenching process. Figure 1a shows the SEM image of mesoporous perovskite PTO fibers, which were characterized to be tens of micrometers in length and 200–500 nm in diameter with good dispersion. A typical PTO fiber in Fig. 1b exhibits regular facets, maintaining a similar morphology to those of the pre-perovskite PTO fibers (Supplementary Figure 1). SAED (Selected area electron diffraction) patterns of different areas on a typical fiber suggest that the mesoporous PTO fiber adopt a single-crystal and single-domain structure (Supplementary Figure 2), where the ferroelectric polarization is along the axial direction of the fibers. Besides, cross-section SEM image in Fig. 1c presents the inner structure of a single perovskite PTO fiber, which is enclosed with many mesopores with a size of 5–10 nm. Accordingly, Fig. 1d illustrates a schematic of a single mesoporous PTO fiber. To further investigate microstructure of the mesopores, cross-section images along radial (the blue plane marked as 1 in Fig. 1d) and axial (the purple plane marked as 2 in Fig. 1d) directions of the fibers were measured by TEM, respectively. Figure 1e, f show that these mesopores with a size of 5–10 nm are randomly distributed within PTO fibers. More importantly, the corresponding SAED image (Fig. 1e, inset) and Fast Fourier Transformation (FFT) pattern (Fig. 1f, inset) show bright and sharp spots along [010] and [001] zone axis, respectively, indicating a single-crystal character of the fiber with an axial direction along [001] tetragonal perovskite PTO. In addition, several typical mesopores were characterized in detail by cross-section HRTEM (Fig. 1g) to exhibit a truncated square shape with a side length of ~5 nm. On the basis of above results, a truncated cube model could be constructed to describe the three-dimensional morphology of mesopores (Fig. 1h), which is basically constituted by the (100), (010), and (001) facets as the boundaries. In addition, BET pore size analysis indicates that the size distribution of mesopores in PTO fibers are peaked 4.7 and 7.5 nm (Supplementary Figure 3), consistent with TEM characterization.

### Thermal expansion performance and ferroelectricity

**Fig. 2:**
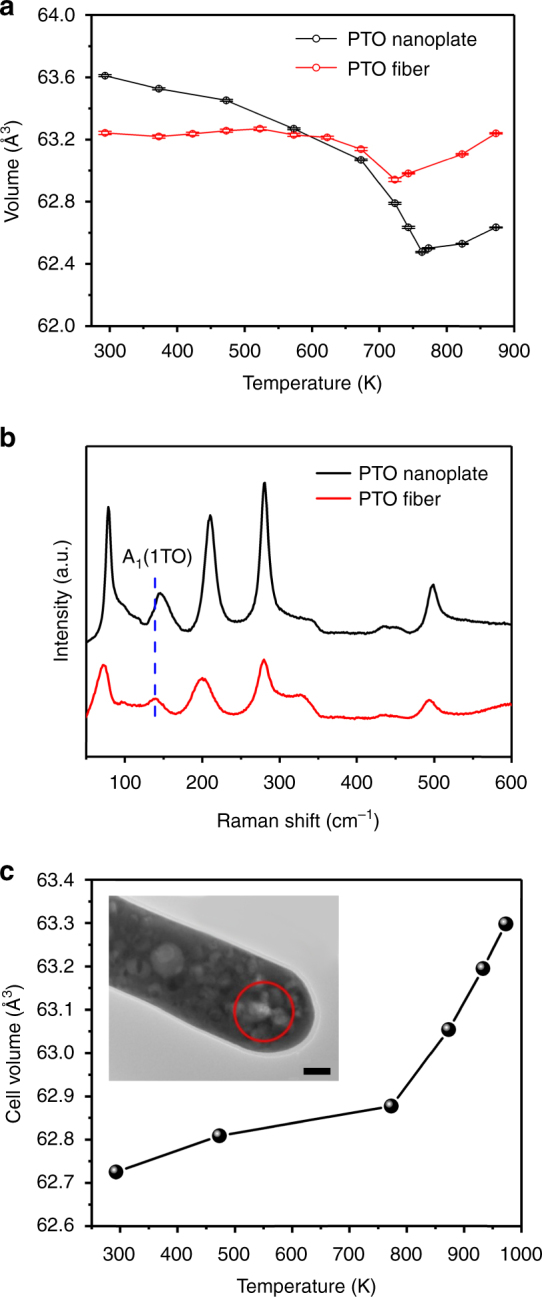
The thermal expansion performance and Raman characterization of mesoporous PTO fibers and porous-free PTO nanoplates. **a** Temperature evolution of cell volume calculated from in situ XRD results. The error bars are derived from the standard deviation of the Voigt profile function, which are employed in the Maud 2.55 software to fit the XRD peaks in Supplementary Figure 5. **b** Raman spectra of mesoporous PTO fibers and porous-free PTO nanoplates, **c** temperature evolution of cell volume corresponding to the microregion marked by a red circle in the inset derived from in situ SAED results. The inset shows the overall TEM image of the considered mesoporous PTO fiber with a diameter of ~80 nm. Scale bar, 20 nm

As shown in Fig. 1, large-scale faceted mesopores were embedded in the single-crystal PTO fibers, making them highly attractive for exploring novel physical properties. Herein, we choose single-crystal and porous-free perovskite PTO nanoplates^24^ as comparisons (Supplementary Figure 4), which behave similar to bulk PTO. The cell volumes of mesoporous PTO fibers and porous-free PTO nanoplates were derived from a Rietveld refinement of in situ XRD patterns (Supplementary Figure 5). It is very interesting to find that the mesoporous PTO fibers show a ZTE performance (Fig. 2a, red pattern) with a (volumetric) TEC of −1.41 × 10^−6^ K^−1^ in a wide temperature range from 293 to 623 K. Note, the systematic error of Rietveld refinement could lead an uncertainty of TEC varying between −0.48 × 10^−6^ K^−1^ and −2.33 × 10^−6^ K^−1^. Nevertheless, it is in the same order of previously reported TEC of ZTE materials with doping (TEC, −1.1 × 10^−6^ K^−1^, 298–403 K)^18^ and solid solutions (TEC, −4.6 × 10^−6^ K^−1^, 298–793 K)^17^. In contrast, PTO nanoplates show a typical NTE performance (Fig. 2a, black pattern) with a TEC of −1.92 × 10^−5^ K^−1^ from 293 to 573 K, in which temperature range the mesoporous PTO fibers demonstrated even more impressive TEC of −6.98 × 10^−7^ K^−1^. Near *T*_c_, the ZTE of PTO fibers and NTE of PTO nanoplates disappear and above *T*_c,_ both of them exhibit PTE with a similar TEC of 3.37 × 10^−5^ K^−1^ and 4.25 × 10^−5^ K^−1^ (823–873 K), respectively. It has been widely discussed that NTE behavior is highly related to the physical properties of the materials, such as magnetism and electronic phase transition^5–10^. In the previous reports, the origin of NTE in ferroelectric materials has been investigated to be closely related to the ferroelectric soft mode^19^. In the case of ferroelectric PTO, the spontaneous ferroelectric polarization at room temperature originates from a large displacement of Ti ions, stabilized by a covalent bonding of Pb–O^25^. On the other hand, the displacement of Ti ions corresponds to the soft-mode motions^26^. Accordingly, a reduced ferroelectric polarization would affect the soft-mode motion, for example, the A_1_(1TO) mode, and thus lead to a reduced NTE. Hence, we investigate the ferroelectricity of PTO fiber to understand the observed ZTE by exploring the tetragonality and soft phonon modes.

Figure 2b shows the Raman spectra of mesoporous PTO nanoplates and PTO fibers, respectively, where a Raman shift of A_1_(1TO) soft mode^27,28^ from 144.8 to 138.6 cm^−1^ could be observed. This result implies a decreased ferroelectricity of mesoporous PTO fibers, supported by a reduced *c*/*a* ratio from 1.064 to 1.047 (Supplementary Figure 6) and a reduced *T*_c_ from ~770 to ~759 K (Supplementary Figure 7) compared with PTO nanoplates. Importantly, the ferroelectric polarization of mesoporous PTO fibers remains a high level since the calculated *c*/*a* is 1.047^29^ (Supplementary Figure 6). On the basis of above results, we argue that the ferroelectricity of PTO fibers reduces slightly and cannot lead to a disappearance of NTE and then an overall ZTE. This argument could be supported by the previous reports, where the slightly reduced ferroelectricity of PTO could only lead to a reduced NTE performance^17,19^. Moreover, an in situ TEM experiment (Fig. 2c and Supplementary Figure 8) was employed to investigate a variation of lattice parameters in a microregion with large amount of mesopores (a circular region with a diameter of 50 nm, marked in the inset of Fig. 2c), and it provides a direct evidence to confirm a PTE performance (with a TEC of ~5.05 × 10^−6^ K^−1^, 273–773 K). These facts allow us to propose that the ZTE of the PTO fibers has an origin from a synergetic effect of NTE arisen from intrinsic ferroelectricity and PTE due to an existence of the mesopores. To examine the influence of mesopores on the thermal expansion property, PTO fibers annealed for different time (5 min, 30 min, and 180 min) were prepared and investigated. TEM images of PTO fibers annealed for 5 min, 30 min, and 180 min indicate that a longer annealing time could lead to less mesopores in the fiber (Supplementary Figure 9). The TEC, as expected, shows a rising sequence of *α*_5min_ (−1.41 × 10^−6^ K^−1^), *α*_30min_ (−1.52 × 10^−6^ K^−1^), and *α*_180min_ (−6.25 × 10^−6^ K^−1^) (293–623 K, Supplementary Figure 10c), indicating the PTO fibers with more mesopores show an improved ZTE performance. Correspondingly, PTO fibers annealed for 5 min, having the largest number of mesopores, exhibit a lowest tetragonality and ferroelectricity (*c*/*a* ~1.047, Raman shift of A_1_(1TO), 138.6 cm^−1^, 293 K), whereas PTO fibers annealed for 180 min exhibit the highest one (*c*/*a* ~1.054, Raman shift of A_1_(1TO), 141.6 cm^−1^, 293 K) (Supplementary Figure 10). Evidently, the thermal expansion property and ferroelectricity of PTO fibers is highly related to the existence of the mesopores. It becomes very appealing to elucidate what happened within the mesopores, leading to the PTE behavior and the reduced ferroelectricity of the fibers.

### Microstructure characterization near a single mesopore

**Fig. 3:**
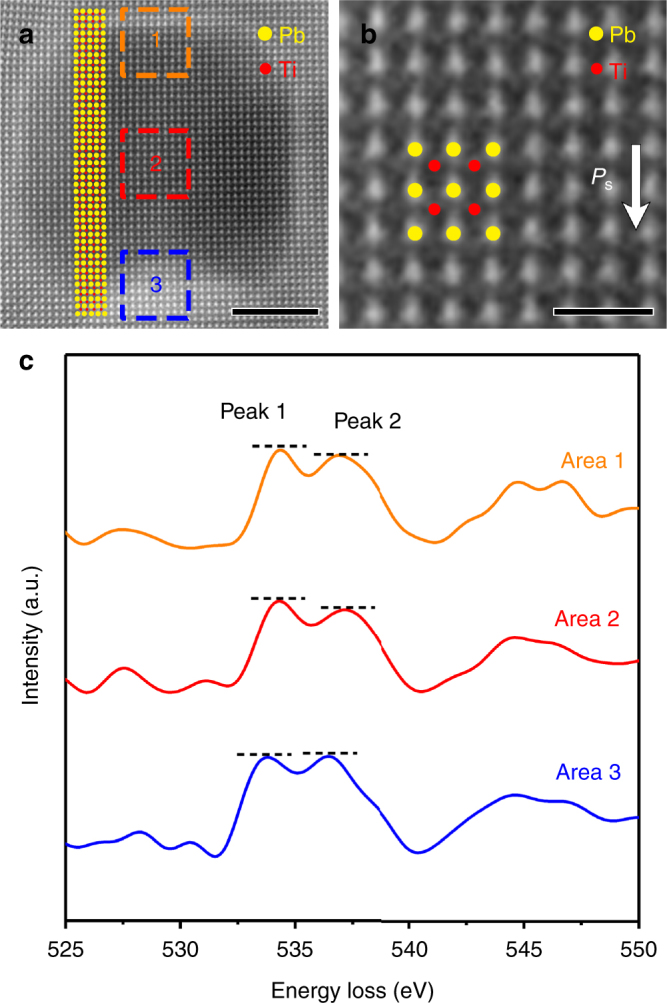
High-resolution HAADF-STEM and EELS characterization of the microregion near a single mesopore. **a** High-resolution HAADF-STEM image of a typical mesopore of mesoporous PTO fibers. Scale bar, 5 nm. **b** Zoom-in image corresponding to area 2 in **a**. Scale bar, 2 nm. The positions of Pb and Ti atoms were marked in the image using yellow and red dots, respectively. **c** EELS spectra of O K edges measured in area 1–3 in **a**, respectively

**Fig. 4:**
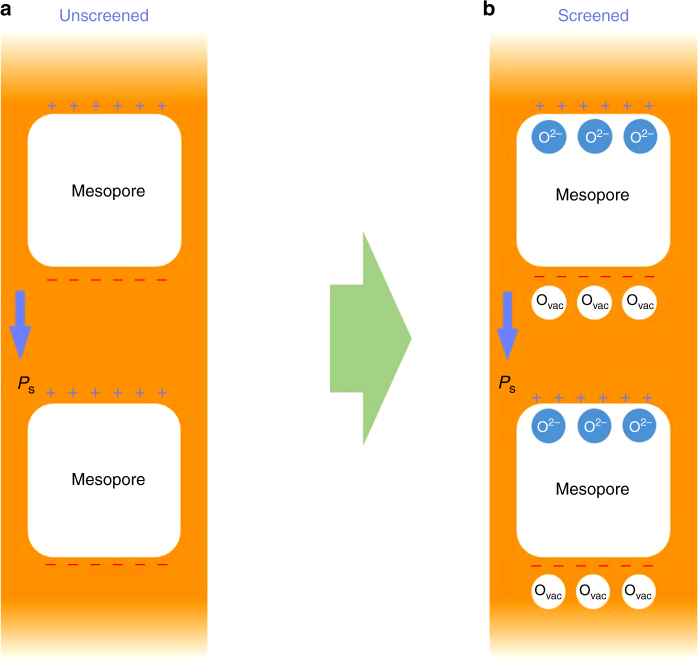
Scheme illustration of oxygen-based polarization screening mechanism in mesopores. **a** Unscreened mesoporous PTO fiber. **b** Screened mesoporous PTO fiber

Figure 3a shows the high-angle annular dark-field scanning transmission electron microscopy (HAADF-STEM) image of a typical mesopore with a size of ~12 nm, showing a truncated cube morphology, which is consistent with Fig. 1g, h. By marking the atom position of Pb (yellow dots) and Ti (red dots), a unified upward displacement of Ti atom was determined, indicating a uniform ferroelectric polarization across the whole mesopore along (001) direction (*c* axis) of perovskite PTO^25^ (Fig. 3a–b), which was further supported by the observation of other mesopores on the same fiber (Supplementary Figure 11). Accordingly, the upper exposed surface of the mesopore should be a positive polar surface and the lower exposed surface is correspondingly the negative one^25^ because of the downward polarization direction. This result agrees well with the pore model in Fig. 1h, where two exposed facets are polar (001) plane along (001) direction. If the exposed polar surfaces are not screened, the surface charge induced by ferroelectric polarization will lead to a strong electric field in the mesopores (Fig. 4a), which would make the system very unstable. Therefore, a ferroelectric polarization screening is necessary^30^.

## Discussion

According to the experiment results, the mesopores were enclosed within the fibers, and no molecular or ionic absorptions^31,32^ from outside are accessible to the polar surfaces for the screening. For a single-domain ferroelectric perovskite oxide, a high concentration electron (~10^20^ cm^−3^) or oxygen vacancy accumulated near the surface is needed for screening when exposed to a vacuum-like environment^30^. In particular, oxygen vacancies have been demonstrated to be able to stabilize the negative polar surface of PTO^33,34^. More interestingly, oxygen vacancy-controlled screening at the BFO/La_x_Sr_1-x_MnO_3_(LSMO) interface (negative polarization surface) has been well established, giving rise to a decrease in Mn vacancy and change in the oxygen K-edge intensity^34^. In the case of PTO fibers, an oxygen-based polarization screening mechanism is presumably proposed (Fig. 4b). On the negative polar surface, the ferroelectric polarization could be screened by the existence of positively charged oxygen vacancies. On the other hand, the screening on the positive polar surface of mesopores could be realized by the accumulation of negative charged oxygen ions (O^2−^).

Such screening mechanism is supported by the electron energy-loss spectroscopy (EELS) spectra of O K edges in Fig. 3c, where the orange, red, and green spectra correspond to area 1–3 in Fig. 3a. As reported, the peak located at ~537 eV (marked as peak 2) in O K edge is closely related to the bonding state between oxygen and the surrounding cations^35,36^, and the strengthened peak 2 represent the appearance of oxygen vacancies^37^. According to Fig. 3c, it can be observed that red and green spectra exhibit a slightly lower peak 2 compared with the peak located at ~534 eV (marked as peak 1), similar to the calculated results of PTO^37^. Differently, the orange spectrum exhibits an obviously strengthened peak 2, which is comparable with peak 1. This result provides the evidence to an appearance of oxygen vacancies in area 3 (near the negative polar surface of the mesopore), while no obvious difference has been observed in area 1 (near the positive polar surface of the mesopore) and area 2 (across the side surfaces), similar to the typical PTO^37^. Meanwhile, an existence of Ti^3+^ near negative polar surface could be observed compared with that near positive polar surface and across the side surfaces (Supplementary Figure 12), possibly induced by the appearance of oxygen vacancies. In addition, energy dispersive X-ray spectroscopy (EDX) linear scan across the mesopore indicates an increased and decreased oxygen concentration on the two surfaces perpendicular to the polarization direction, respectively (Supplementary Figure 13). Further EDX linear scan parallel and vertical to the ferroelectric polarization (*P*_s_) direction suggests that the oxygen concentration is polar surface-dependent (Supplementary Figure 14). On the basis of the above discussion, we argue that the polarization screening within the mesopores is realized by the existence of oxygen vacancies on negative polar surface and the accumulation of oxygen on positive polar surface. Accordingly, the polarization screening via oxygen vacancies would lead to a disappearance of ferroelectricity within the near surface layers of the mesopores^30^ and thus the PTE (Fig. 2c) in the microregion of PTO fibers.

In summary, by introducing large-scale mesopores, ZTE in a relatively high and wide temperature range (TEC, −1.41 × 10^−6^ K^−1^, 293–623 K) has been achieved in single-phase PTO fibers. The mesopores with sizes of 5–10 nm are characterized to be truncated cube in morphology and enclosed within the fibers. Particularly, the pores were distributed in the uniform ferroelectric polarization with two polar surface exposed, which was probably screened by an appearance of oxygen vacancies on negative polar surface and an accumulation of oxygen on positive polar surface. The polarization screening could give rise to the disappearance of ferroelectricity near the mesopores. The ZTE performance of PTO fibers is attributed to a synergetic effect of NTE from intrinsic ferroelectricity and PTE due to the polarization screening near the mesopores. These findings suggest a fascinating strategy to fabricate single-phase ZTE materials by building fascinating ferroelectric surfaces in PTO-based ferroelectrics.

## Methods

### Materials

Mesoporous PTO fibers were synthesized by a two-step process. Firstly, the pre-perovskite PTO fibers were prepared via a polymer-assisted hydrothermal method. Analytically pure tetrabutyl titanate [(C_4_H_9_O)_4_Ti] and lead nitrate Pb(NO_3_)_2_ were used as raw materials, while potassium hydroxide (KOH) and polyvinyl alcohol (PVA) were used as mineralizer and surfactants, respectively. The specific steps are described as follows: Tetrabutyl titanate [(C_4_H_9_O)_4_Ti, 1.736 g] was dissolved in ethylene glycol monomethyl ether (10 ml) to get a solution, where 3–5 ml ammonia was added to obtain the white Ti(OH)_4_ precipitate as the Ti precursor. Then the as-prepared Ti precursor was washed with deionized water for several times in order to remove the ammonium ions. Meanwhile, moderate Pb(NO_3_)_2_ were dissolved in deionized water to obtain the Pb precursor, adjusting the atomic ratio of Pb/Ti to 1.1. Finally, the Pb and Ti precursors, KOH (1.37 g) and PVA aqueous solution (0.8 g L^−1^) were mixed and stirred for 1 h. Deionized water was then added to adjust the total volume of the solution to 45 ml. The resulting precursor suspension was moved into a 50 ml stainless-steel Teflon-lined autoclave for the hydrothermal treatment at 200˚C for 10 h. Finally, it was cooled down to room temperature naturally. To remove the residue from the hydrothermal reaction, the as-prepared samples were washed with deionized water and ethanol for several times. The washed products were dried in oven at 80 ˚C for 12 h to obtain the pre-perovskite PTO fibers. Subsequently, the as-prepared pre-perovskite PTO fibers were placed in crucibles and annealed at 600 °C for 5 min (30 min or 180 min) in a furnace, followed by a rapid cooling in the air, resulting in mesoporous PTO fibers.

### Characterization

The room temperature and in situ XRD data were collected on a PANalytical Empyrean powder diffractometer by Cu K_α_ radiation (*λ* = 1.54056 Å). For the in situ XRD measurement, the powder sample was maintained at a specified temperature (293–873 K) for 15 min to reach heat equilibrium. The scanning speed of 2*θ* angle was 10° min^−1^ and the heating speed was 2 K min^−1^. The lattice constants and cell volume values were derived from the XRD patterns by the Rietveld method in Maud 2.55 software using a tetragonal (P4mm) model. The morphology and microstructure of the samples were characterized by SEM (Hitachi SU-70), TEM (FEI Tecnai G^2^ F20 S-TWIN), and aberration-corrected HAADF-STEM (FEI Titan G^2^ 80-200 Chemi STEM), respectively. The STEM-EELS line scan of fine Ti L edge was performed using a pixel time of 700 ms and a dispersion of 0.05 eV per pixel (corresponding energy resolution, 0.8 eV). The O K edge spectra was conducted with an exposure time of 700 ms per pixel and a dispersion of 0.1 eV per pixel (corresponding energy resolution, 1.5 eV). The spectra were then smoothed using the low-pass filters in DigitalMicrograph Software. The in situ TEM characterization was performed on Hitachi H9500, where the heating speed was set as 10 K min^−1^. The sample was maintained at a specific temperature (293, 473, 773, 873, 933, and 973 K) for 5 min to reach heat equilibrium. Raman spectra were acquired using a 100× objective on a Renishaw InVia Raman microscope equipped with an ultralow noise charge-coupled device detector at room temperature. TG-DSC curves were acquired using SDT Q-600 simultaneous thermal analyzer.

### Data availability

The data that support the findings of this study are available from the corresponding author upon request.

## Electronic supplementary material

Supplementary Information
