# Supplementary material for: Mesopores induced zero thermal expansion in single-crystal ferroelectrics
Source: Nat Commun. 2018 Apr 24;9:1638. doi: 10.1038/s41467-018-04113-y (PMC5915410; doi:10.1038/s41467-018-04113-y)
Supplement: Supplementary file 1 — Supplementary Information [file 41467_2018_4113_MOESM1_ESM.pdf]

Supplementary Information for

Mesopores induced zero thermal expansion in single-crystal ferroelectrics

Ren et al.

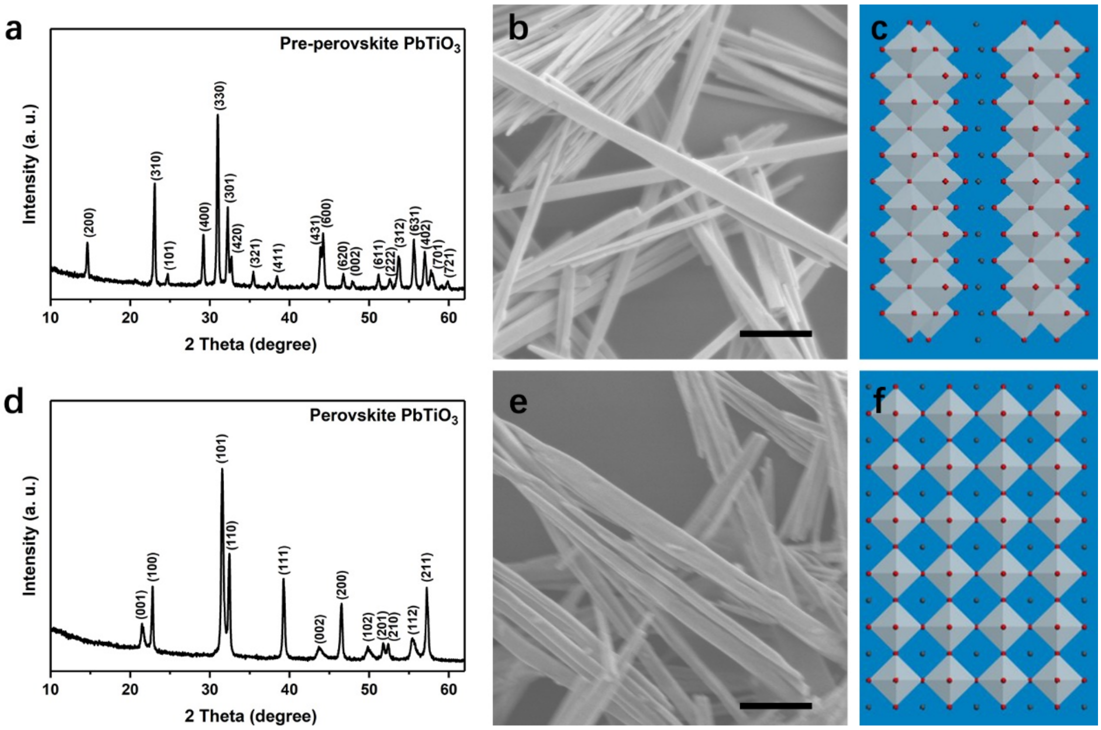

Supplementary Figure 1 Morophology and microstructure characterization of pre-perovskite PTO and mesoporous perovskite PTO fibers. **a**,**d** XRD patterns and **b**,**e** SEM images of pre-perovskite PTO and mesoporous perovskite PTO fibers, respectively. Scale bar, 1 μm. **c**,**f** Illustration of pre-perovskite and perovskite PTO microstructure, respectively. The red spheres and black spheres represent O atoms and Pb atoms, respectively.

According to the X-ray diffraction (XRD) patterns (Supplementary Figure 1a, 1d), the as-prepared pre-perovskite and perovskite $PbTiO_3$ (PTO) fibers are characterized to be single-crystal and single-phase. The scanning electron microscopy (SEM) images demonstrate that the overall morphologies of pre-perovskite and perovskite fibers are quite similar (Supplementary Figure 1b, 1e). This result indicates that the pre-perovskite PTO fibers have completely transformed into perovskite PTO fibers, maintaining their original morphology.

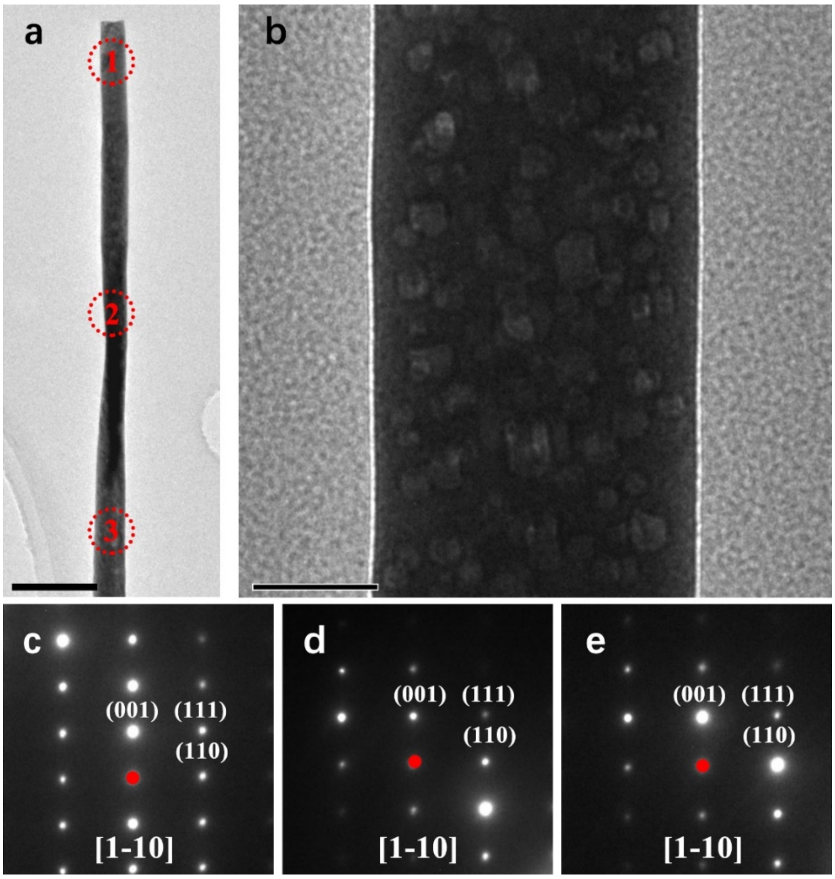

Supplementary Figure 2. TEM and SAED characterization of a single mesoporous PTO fiber. **a-b** TEM images of a typical mesoporous PTO fiber. Scale bar, 500 nm and 50 nm, respectively. **c-e** the SAED patterns corresponding to the area 1-3 marked using red dashed circle in **a**, respectively. The red points correspond to the center points of the diffraction, respectively.

Supplementary Figure 2a demonstrates the morphology of the fibers with a diameter of ~100 nm, and Supplementary Figure 2b verifies their mesoporous structure. Furthermore, three areas of the fiber were chosen to obtain their SAED patterns, where clear and sharp diffraction spots could be detected in Supplementary Figure 2c-e. It is worth noting that the three diffraction patterns of each fiber show the same orientation, and no splitting of the diffraction spots could be observed, implying that the three areas of each fiber has a single-domain structure or uniform polarization direction.

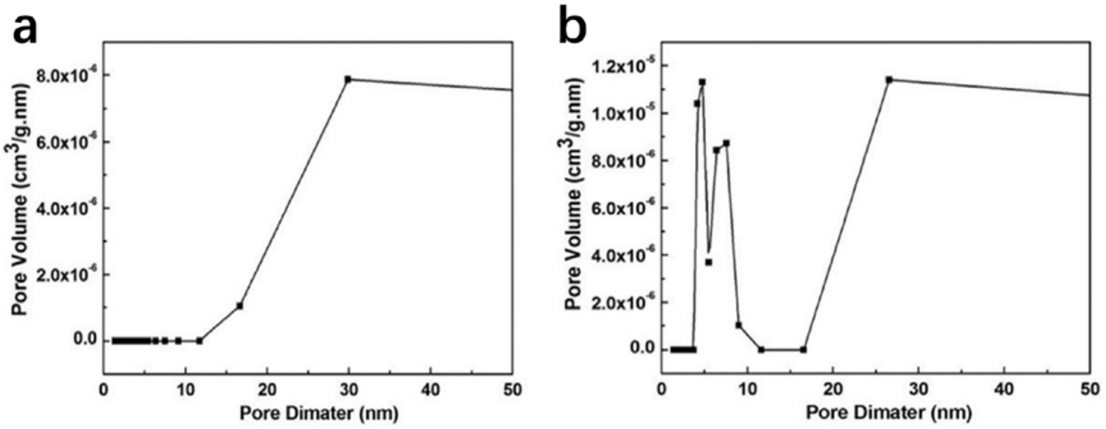

Supplementary Figure 3. BET pore diameter charaterization of the mesoporous PTO fibers before and after acid etching. **a** As-prepared mesoporous PTO fibers. **b** 10% nitric acid etching mesoporous PTO fibers, showing the mesopores are enclosed with the size mainly distributed in 4.7 nm and 7.5 nm

Compared with as-prepared PTO fibers, the 10% nitric acid etching PTO fibers exhibit a large number of mesopores with the size mainly distributed in 4.7 nm and 7.5 nm due to the surface corrosion induced exposure of the inner mesopores, which strongly supports that the mesopores are enclosed within the PTO fibers.

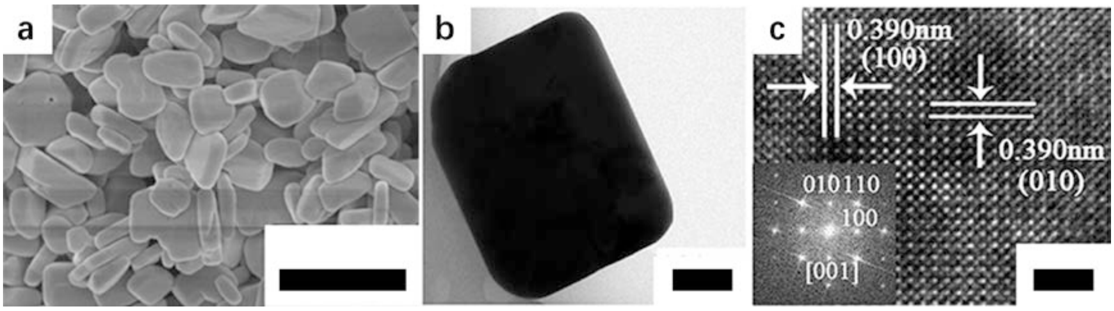

Supplementary Figure 4. Morphology and microstructure characterization of PTO nanoplates. **a** SEM image (scale bar, 1 μm), **b** TEM image (scale bar, 200 nm) and **c** HRTEM image (scale bar, 2 nm) of single-crystal perovskite PTO nanoplates. The PTO nanoplates exhibit a well-defined structure with a rectangular outline and a side length of ~800 nm. The spacing of lattice fringes is characterized to be 0.390 nm and 0.390 nm, corresponding to (100) and (010) of perovskite PTO, respectively.

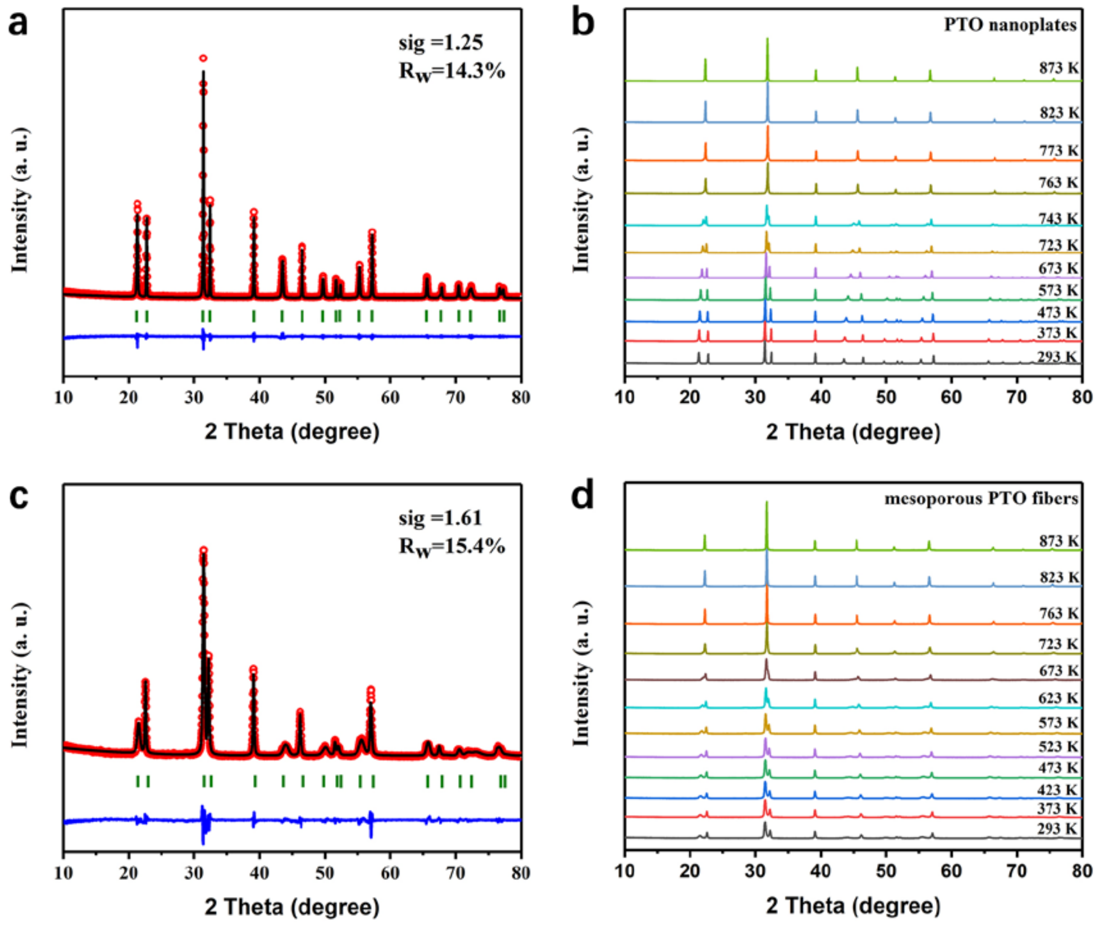

Supplementary Figure 5 Rietveld full profile refinement and *in-situ* XRD patterns of the PTO nanoplates and mesoporous PTO fibers. **a,c** XRD pattern of PTO nanoplates and mesoporous PTO fibers at room temperature (293 K). The red circle and black line represent the observed and refined X-ray powder diffraction results, respectively. The blue solid line exhibits the difference profile, and the green marks show the reflection positions. **b**,**d** *In-situ* XRD patterns of PTO nanoplates and mesoporous PTO fibers at different temperatures, respectively.

Rietveld structure refinement of Bragg peaks was undertaken with the tetragonal P4mm space group using Maud 2.55 software. The calculated results of cell parameter in Figure 2a were derived from the XRD patterns in Supplementary Figure 5b and d.

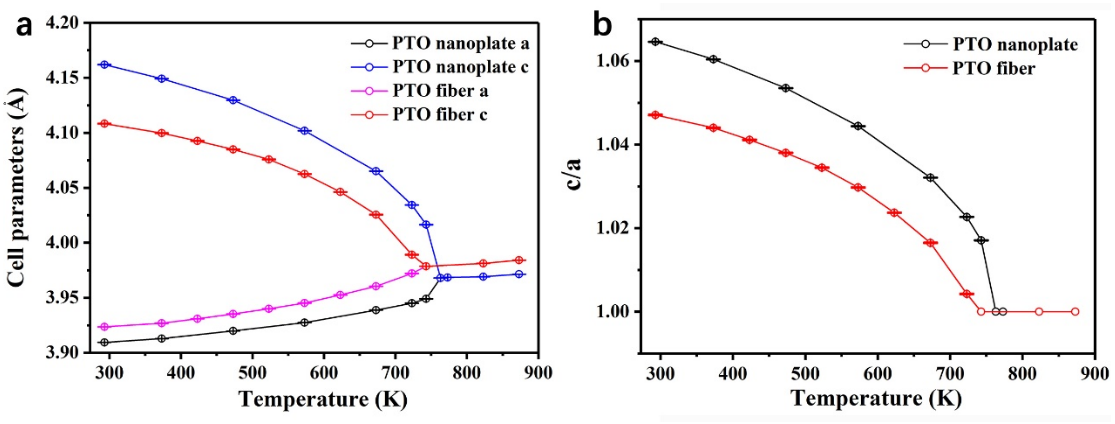

Supplementary Figure 6 Cell parameters of the PTO nanoplates and mesoporous PTO fibers. **a** Cell parameters and **b** *c*/*a* of porous-free PTO nanoplates and mesoporous PTO fibers derived from *in-situ* XRD results in Supplementary Figure 5b. The error bars are derived from the standard deviation of the Voigt profile function, which are employed in the Maud 2.55 software to fit the XRD peaks in Supplementary Figure 5.

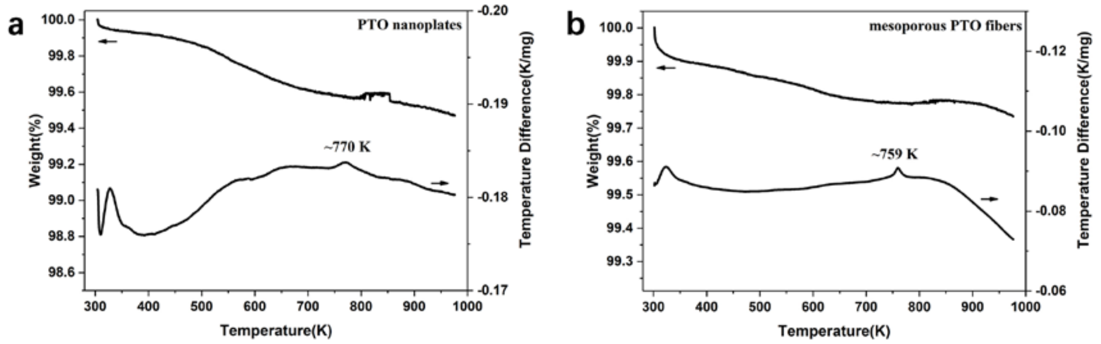

Supplementary Figure 7 TG-DSC characterization of PTO nanoplates and mesoporous PTO fibers. **a** TG-DSC curves of PTO nanoplates. **b** TG-DSC curves of mesoporous PTO fibers. These results exhibit a Curie temperature of ~770 K (PTO nanoplates) and ~759 K (mesoporous PTO fibers), respectively.

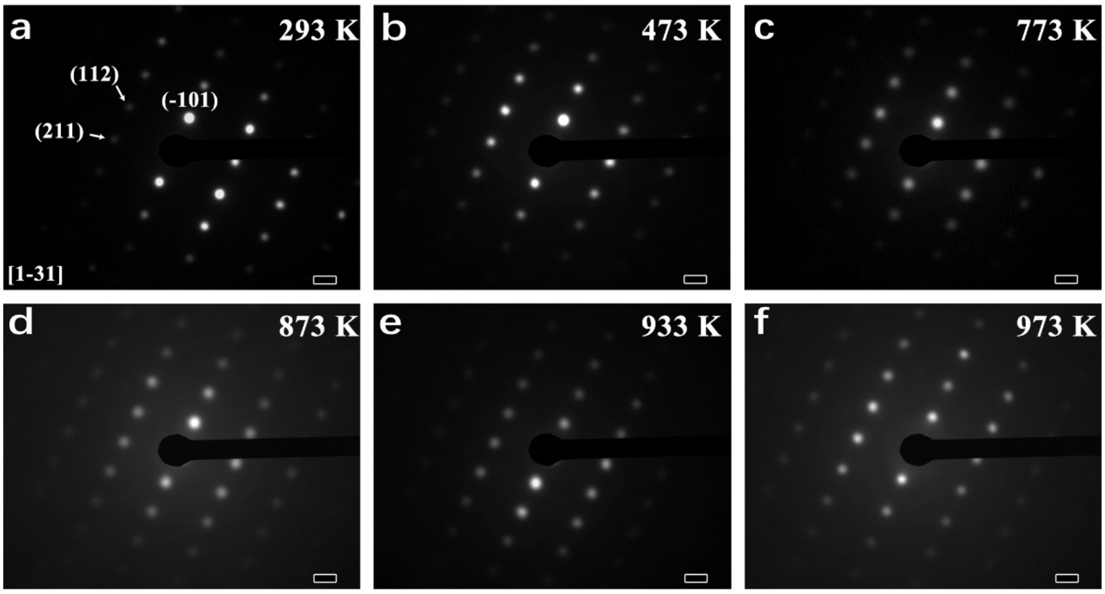

Supplementary Figure 8 Selected area electron diffraction (SAED) characterization of a single mesoporous PTO fiber. **a-f** *In-situ* SAED patterns measured at 293 K, 473 K, 773 K, 873 K, 933 K and 973 K, respectively. Scale bar, 5 $Å^{-1}$. The selected area (with a diameter of 50 nm) are marked using a red circle in Figure 2c inset, which is on a typical mesoporous PTO fiber.

The diffraction spots in Supplementary Figure 6a-f are bright and sharp with no splitting, indicating a single-crystal and single-domain character of the selected area. Based on the diffraction pattern, the cell volume has been calculated and the result is shown in Figure 2c.

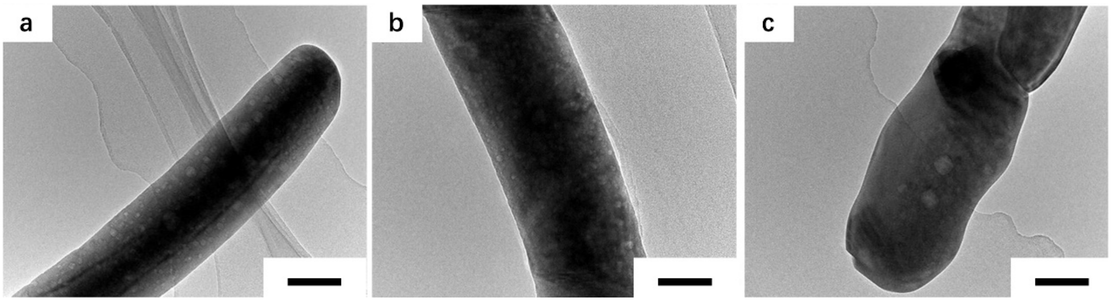

Supplementary Figure 9 TEM images of mesoporous PTO fibers prepared for differnt annealing time. **a** 5 min. **b** 30 min. **c** 180 min. The result shows the controllable amount of the mesopores by adjusting the annealing time. Scale bar, 100 nm.

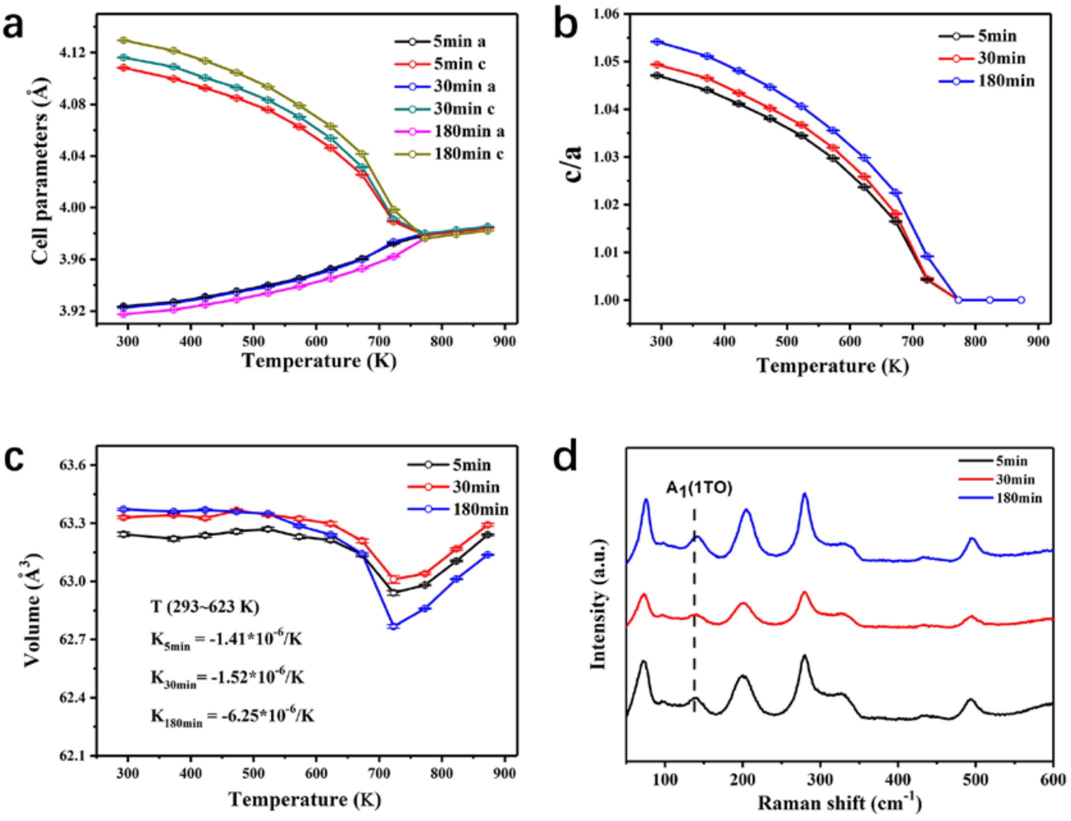

Supplementary Figure 10 The thermal expansion performance and Raman characterization of mesoporous PTO fibers prepared for different annealing time. Temperature evolution of **a** cell parameters, **b** *c*/*a* and **c** cell volume of mesoporous PTO fibers prepared for the annealing time of 5min, 30 min and 180 min. The error bars are derived from the standard deviation of the Voigt profile function, which are employed in the Maud 2.55 software. The TEC of these three samples are listed in **c**, indicating the PTO fibers synthesized at a shorter annealing time show an improved ZTE performance. **d** Raman spectra of mesoporous PTO fibers prepared for the annealing time of 5min, 30 min and 180 min. The A_1(1TO) peaks of the 5 min, 30 min and 180 min samples are located at 138.6 cm^{-1}, 140.1 cm^{-1} and 141.6 cm^{-1}, suggesting an enhanced ferroelectricity by the prolonging of the annealing time.

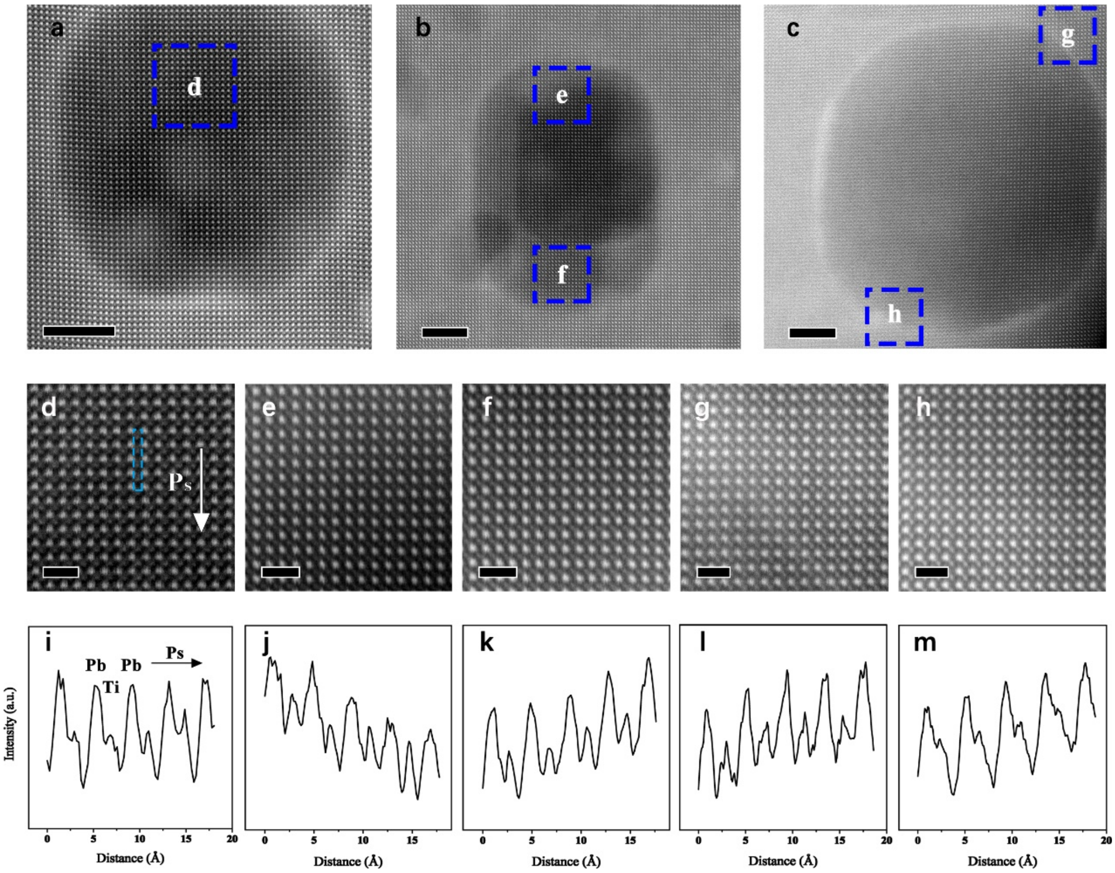

Supplementary Figure 11 HAADF-STEM characterization near the mesopores. **a-c** HAADF-STEM images of three typical mesopores in PTO fibers. Scale bar, 5 nm. **d-h** zoom-in images of five microregions marked using blue dashed rectangles in a-c. Scale bar, 1 nm. **i-m** the corresponding profile intensity patterns of four random cells in **d-h** along c-axis.

To further verify the uniform polarization near the mesopores, three typical mesopores with different size on a same fiber were selected to investigate the displacement of Ti atom (Supplementary Figure 11a-c). The zoom-in images of five microregions marked using blue dashed rectangles in Supplementary Figure 11a-c were shown in Supplementary Figure 11d-h, respectively, and the Supplementary Figure 11i-m shows the corresponding profile intensity patterns of four random cells along c-axis (for example, four cells marked using a blue dashed rectangle in Supplementary Figure 11d, from top to bottom). Due to the higher atomic weight, the peak intensity

of Pb atom is higher than that of Ti atom, which is marked in Supplementary Figure 11i. According to the patterns in Supplementary Figure 11i-m, the Ti peak is always located at the middle left of two Pb peaks, and thus an upper displacement of Ti atoms and a downward polarization direction can be determined (marked as P_s in Supplementary Figure 11d). These results suggest that the polarization direction near the mesopore is uniform, agreeing well with the results in our manuscript.

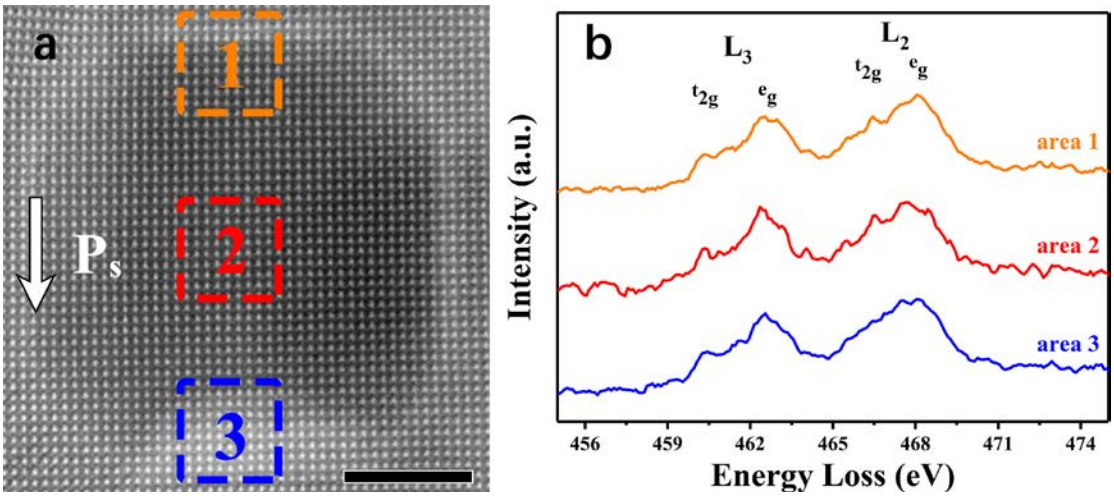

Supplementary Figure 12 EELS charaterization of Ti element near the mesopore. **a** High-resolution HAADF-STEM image of a typical mesopore with a size of ~12 nm. Scale bar, 5 nm. The polarization direction is determined to be down according to Figure 3b (marked in the image). Therefore, the upper exposed surface is positive polar surface while the lower exposed surface is negative polar surface. **b** EELS spectra of Ti L edges measured in area 1, 2 and 3 in **a**, respectively.

The Ti L edge of area 1 (near positive polar surface) and area 2 (across the side surfaces) shows a four-peak configuration, whereas the Ti L edge of area 3 (near negative polar surface) shows three peaks. The combined Ti L_2 t_{2g} peak and Ti L_2 e_g peak in area 3 indicate an existence of Ti^{3+} near the negative polar surface[1], which is possibly attributed to an excess of oxygen vacancies.

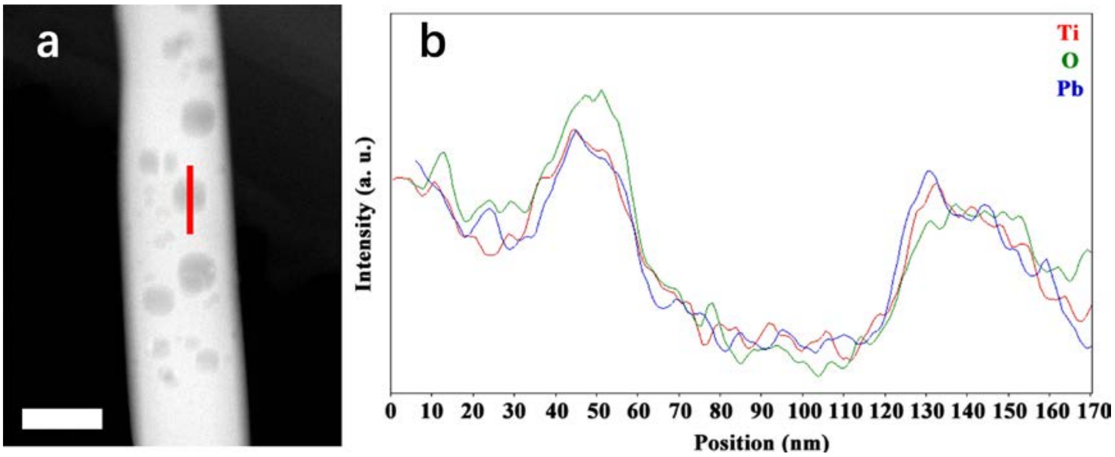

Supplementary Figure 13 EDX liner scanning of a mesopore along c-axis. **a** STEM image of a single mesoporous PTO fiber of the sample annealed for 180 min. The PTO fiber exhibit a diameter of ~220 nm and the considered mesopore shows a size of ~100 nm. Scale bar, 200 nm. **b** Energy dispersive X-ray spectroscopy (EDX) linear scanning results corresponding to the red line along axial direction in **a**, exhibiting an obviously increasement of oxygen concentration on one suface of the mesopore and decreasement on another.

It is hard to identify the tiny variation of the oxygen concentration near the general mesopores (with a size of 5~10 nm) because EDX results reflect the charater across the whole PTO fiber. Therefore, we choose a large mesopore with the size of ~100 nm to perform EDX linear scanning analysis, which could make the pattern clear and convincing.

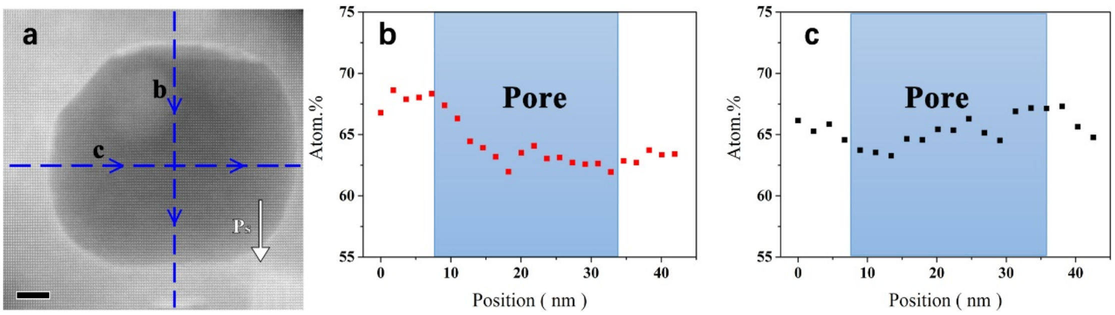

Supplementary Figure 14 Horizontal and vertical EDX profile scanning across one mesopore. **a** HAADF-STEM image of a single mesopore in mesoporous PTO fibers annealed for 180 min. Scale bar, 5 nm. **b-c** the EDX profile scanning results corresponding to the blue dashed line marked as b and c in **a**.

The two profiles were parallel and vertical to the ferroelectric polarization (P_s) direction, respectively (Supplementary Figure 14a). As shown in Supplementary Figure 14b, one can find that oxygen element concentration near the positive polar surface was ~68%, obviously higher than that near the negative polar surface (~63%). In constrast, the concentration of oxygen element randomly fluctuates near ~65% when the profile is vertically to the polarization direction (Supplementary Figure 14c). Based on the result, we suggest that the oxygen concentration is polar surface-dependent.

Reference

[1] Ohtomo, A., Muller, D. A., Grazul, J. L. & Hwang, H. Y. Artificial charge-modulationin atomic-scale perovskite titanate superlattices. *Nature* **419**, 378-380 (2002).
